# Field Evaluation of a Safe, Easy, and Low-Cost Protocol for Shipment of Samples from Suspected Cases of Foot-and-Mouth Disease to Diagnostic Laboratories

**DOI:** 10.1155/2023/9555213

**Published:** 2023-08-05

**Authors:** Aurore Romey, Hussaini G. Ularamu, Abdulnaci Bulut, Syed M. Jamal, Salman Khan, Muhammad Ishaq, Michael Eschbaumer, Graham J. Belsham, Cindy Bernelin-Cottet, Anthony Relmy, Mathilde Gondard, Souheyla Benfrid, Yiltawe S. Wungak, Claude Hamers, Pascal Hudelet, Stéphan Zientara, Labib Bakkali Kassimi, Sandra Blaise-Boisseau

**Affiliations:** ^1^Animal Health Laboratory, Foot-and-Mouth Reference Laboratory, Virology JRU, ANSES, INRAE, ENVA, Paris-Est University, Maisons-Alfort 94700, France; ^2^FMD Research Centre, National Veterinary Research Institute (NVRI), PMB 01 Vom, Lagos, Nigeria; ^3^SAP/FMD Institute, Dumlupinar Bulvard 35, Ankara 06510, Turkey; ^4^Department of Biotechnology, University of Malakand, Chakdara 18800, Khyber Pakhtunkhwa, Pakistan; ^5^Institute of Diagnostic Virology, Friedrich-Loeffler-Institut, Greifswald-Insel Riems, Greifswald 17489, Germany; ^6^DTU National Veterinary Institute, Technical University of Denmark, Lindholm, Kalvehave DK-4771, Denmark; ^7^Department of Veterinary and Animal Sciences, University of Copenhagen, Frederiksberg C 1870, Denmark; ^8^The Veterinary Public Health Center, Boehringer Ingelheim Animal Health, 29 Avenue Tony Garnier, Lyon, 69007, France

## Abstract

Identification and characterization of the foot-and-mouth disease virus (FMDV) strains circulating in endemic countries and their dynamics are essential elements of the global FMD control strategy. Characterization of FMDV is usually performed in reference laboratories (RL). However, shipping of FMD samples to RL is a challenge due to the cost and biosafety requirements of transportation, resulting in a lack of knowledge about the strains circulating in some endemic areas. In order to simplify this step and to encourage sample submission to RL, we have previously developed a low-cost protocol for the shipment of FMD samples based on the use of lateral flow devices (LFDs) combined with a simple virus inactivation step using 0.2% citric acid. The present study aimed to evaluate this inactivation protocol in the field. For this purpose, 60 suspected FMD clinical samples were collected in Nigeria, Pakistan, and Turkey, three countries where FMD is endemic. Sample treatment, testing on LFDs, and virus inactivation steps were performed in the field when possible. The effectiveness of the virus inactivation was confirmed at the RL. After RNA extraction from the 60 inactivated LFDs, all were confirmed as FMDV positive by real-time reverse transcription polymerase chain reaction (RT-PCR). The serotype was identified by conventional RT-PCR for 86% of the samples. The topotype and/or lineage was successfully determined for 60% of the samples by Sanger sequencing and sequence analyses. After chemical transfection of RNA extracted from inactivated LFDs, into permissive cells, infectious virus was rescued from 15% of the samples. Implementation of this user-friendly protocol can substantially reduce shipping costs, which should increase the submission of field samples and therefore improve knowledge of the circulating FMDV strains.

## 1. Introduction

Foot-and-mouth disease (FMD) is a contagious transboundary animal disease [1, 2], which causes significant economic damage in both developed and developing countries [3, 4]. It is considered a priority notifiable infectious disease by World Organisation for Animal Health [5]. FMD affects wild and domestic cloven-hooved animals, including cattle, pigs, goats, sheep, deer, and African buffalo [6]. In infected animals, it causes weakness, fever, lameness, and vesicles in and around the mouth, on the muzzle, teats, and feet [7].

FMD is caused by foot-and-mouth disease virus (FMDV) [2, 8], which belongs to the Aphthovirus genus within the family Picornaviridae. The virus exists as seven immunologically distinct serotypes, named A, Asia-1, C, O, SAT 1, SAT 2, and SAT 3. Within each serotype, genomic diversity is classified into genotypes, topotypes (linked to geographical location), lineages, and sublineages [9]. FMD is endemic in Africa, most of Asia, and parts of South America, distributed in seven geographic pools of circulating viruses [10]. In Pakistan, for example, disease outbreaks are caused by FMDV serotypes O, A, and Asia-1 within pool 3 [11]. In Turkey, in Anatolia, serotypes O and A are common [9, 12], and serotype Asia-1 has also been reported [13]. In Nigeria, viruses from pool 5, including serotypes O, A, SAT 1, and SAT 2, have been documented [14–18]. Furthermore, in FMD-free countries, sporadic outbreaks can occur, such as in the United Kingdom, France, and the Netherlands in 2001 [19], again in the United Kingdom in 2007 [20], as well as in Bulgaria in 2011 [21].

Characterization of FMDV strains that are circulating within endemic zones should allow improved disease control by the implementation of vaccination campaigns with the appropriate vaccine strain, thus promoting advancement along the FMD Progressive Control Pathway [22]. Knowledge of emerging strains also serves as an “early warning” of threats to other countries. However, there are still knowledge gaps regarding circulating strains in some endemic areas because of a lack of FMD diagnostic capacity and low frequency of sample submissions to reference laboratories (RL). The lack of submissions is in part related to the high cost of shipping potentially infectious samples while strictly respecting the cold chain and biosafety requirements. Evaluating methods for the preservation of clinical samples at their source in a form that is safe and not prone to degradation during transportation is of great interest. In a previous study, we reported a cost-effective and safe protocol for the shipment of samples from suspected cases of FMD based on the chemical inactivation of FMDV on lateral flow devices (LFDs) used for viral antigen detection [23]. This protocol has been developed and evaluated using reference strains and archival samples of the virus. It allows subsequent detection and typing of FMDV by reverse transcritpion polymerase chain reaction (RT-PCR) and the rescue of infectious FMD virus following RNA transfection into permissive cells.

The present study aimed to further evaluate the performance and the safety of this protocol using freshly collected clinical samples through collaboration with field veterinarians in three endemic countries. More specifically, the entire process was applied directly in the field in Pakistan, Turkey, and Nigeria during FMD outbreaks. In parallel, the same protocol was applied to samples collected from experimentally infected cattle. The results of this study are described here, and the perspectives offered by this procedure are discussed.

## 2. Materials and Methods

### 2.1. Inactivating Solution

Citric acid solution at 1% (w/v) was prepared by dissolving citric acid monohydrate (C_6_H_8_O_7_) (Sigma-Aldrich, Saint-Louis, MO, USA, reference C1909, or Merck, Germany) in sterile distilled water. After 24 hr of incubation at room temperature, a working solution of citric acid solution at 0.2% (w/v) was prepared by diluting the 1% citric acid solution 1 : 5 in water. To study it, the working solution was aliquoted in 15 mL conical tubes and stored at 5 ± 3, 21 ± 5, or 37 ± 3°C. The pH of this solution was measured from an aliquot kept at each of the three storage temperatures over a period of 18 months at 2-week intervals. A final pH was taken 3 years after the preparation of the working solution.

### 2.2. Field Sample Collection and Treatment in the Field

Sixty epithelium samples were collected from suspected clinical cases of FMD in three countries where the disease is endemic: twenty samples from Nigeria were collected between April and July 2018, 20 samples from Turkey were collected during June and July 2018, and 20 samples from Pakistan were collected from January 2017 to April 2019. The location, date of sampling, and host species are listed in *Supplementary 1*.

**Figure 1:**
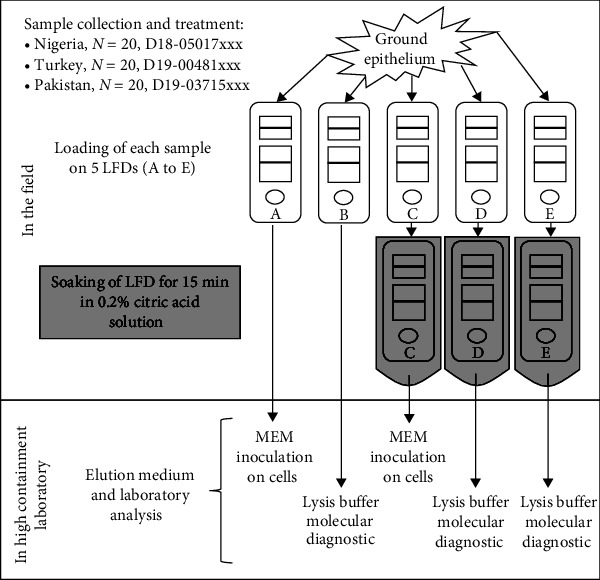
Schematic workflow of the study design applied to field samples collected in Turkey, Nigeria, and Pakistan. Reference and number of processed samples are detailed (xxx = unique sample identification). In Pakistan, epithelium samples were loaded only on LFDs B and D.

In Nigeria and Turkey, samples were directly treated in the field. In Pakistan, after collection, samples were stored in 50% glycerol buffer until treatment in the laboratory. Each epithelium sample was cut into small pieces and transferred into a small tube with sand included in the Svanova® FMDV extraction kit (Boehringer Ingelheim Svanova, Uppsala, Sweden). Samples were ground vigorously in 2 mL of the buffer included in the Svanova® FMDV Antigen detection kit (Boehringer Ingelheim Svanova) according to the manufacturer's instructions. After decanting, 200 *µ*L of supernatant from each ground epithelium sample were loaded onto two (labeled B and D, in Pakistan) or five (labeled A to E, in Nigeria and Turkey) LFDs (Figure 1), included in the Svanova® FMDV Antigen detection kit, according to the manufacturer's instructions. The residual supernatants were stored at <−70°C. LFD results were read after 10 min migration time, and results were confirmed after an additional 20 min of incubation. The LFDs were then processed, as shown in Figure 1. Of the five LFDs (A to E) loaded with each individual sample in Nigeria and Turkey, three LFDs out of five (C, D, and E) were soaked in 0.2% citric acid solution for 15 min. In Pakistan, one (D) of the two LFDs loaded for each sample was soaked in a citric acid solution. The treated LFDs were all labeled “inactivated LFD” (*n* = 140 in total). Other LFDs (labeled A and B), not treated with citric acid, were labeled “non-inactivated LFD” (*n* = 100 in total).

### 2.3. Laboratory Cell Lines and Viruses

Swine kidney epithelial cells (IBRS-2, CCLV-RIE 103; FLI, Riems, Germany) were grown in Earle's minimum essential medium (MEM) with L-glutamine (Invitrogen, Carlsbad, CA, USA), supplemented with 100 U/mL of penicillin–streptomycin (PS) (Invitrogen), 1.5% lactalbumin hydrolysate (Sigma-Aldrich), 25 mM HEPES (Invitrogen) and 7% fetal calf serum (FCS) (Eurobio, Les Ulis, France). Fetal goat tongue epithelial cells (ZZ-R 127, CCLV-RIE 127; FLI) [24] were grown in 45% Iscove's Modified Dulbecco's media (IMDM) with GlutaMax (Invitrogen), 45% DMEM/F-12 (1 : 1 mixture of Dulbecco's modified Eagle's media (DMEM) and Ham's F-12) (Invitrogen), supplemented with 100 U/mL of PS and 10% FCS. Baby hamster kidney fibroblastic cells (BHK-21 clone 13, ECACC, Salisbury, United Kingdom) were grown in Earle's MEM, supplemented with 100 U/mL PS, 1 mM nonessential amino acids (Invitrogen), 1 mM sodium pyruvate (Invitrogen), 2 mM L-glutamine (Invitrogen) and 10% FCS. Porcine kidney epithelial cells expressing bovine integrin (LFBK-*α*V*β*6; Plum Island Animal Disease Center, Orient, NY, USA) [25] were grown in DMEM supplemented with 10% FCS. Cells were grown at 37°C in a 2.5% (ZZ-R 127) or 5% (IBRS-2, BHK-21, and LFBK-*α*V*β*6) CO_2_ atmosphere. They were routinely passaged once (ZZ-R 127) or twice (IBRS-2, BHK-21, and LFBK-*α*V*β*6) per week in 75 cm^2^ tissue culture flasks.

The FMDV reference strain O/IRN/13/2012 (lineage O/ME-SA/PanAsia-2/ANT-10) was provided by the Vesicular Disease Reference Laboratory (The Pirbright Institute, Pirbright, UK). This strain was used for the comparison of different kits and experimental conditions for RNA transfection (detailed in Section 2.10). Additionally, this strain was used as a positive control for all RNA transfections with selected kits and conditions.

Clarified supernatant of homogenized vesicular epithelium from a calf infected with a twice plaque-purified clone of the O/FRA/1/2001 isolate of FMDV from the ANSES laboratory collection was used for the animal experiment (see Section 2.4).

### 2.4. Animal Experiment

Six Holstein heifers were inoculated with 10^8^ TCID_50_ (determined by end-point titration on LFBK-*α*V*β*6 cells) of cattle-passaged O/FRA/1/2001 by intranasopharyngeal deposition [26] of vesicular epithelial homogenate diluted to a final volume of 2 mL in DMEM. For the inoculation and sample collection, the animals were deeply sedated by intramuscular injection of 0.3 mg xylazine per kg body mass into the hindquarters. After each procedure, the sedation was reversed by intramuscular injection of 0.03 mg atipamezole per kg body mass. All inoculated animals developed generalized FMD. Vesicular fluid was collected with a fine needle from lesions in the interdigital cleft at 3-4 days after inoculation. Six vesicular fluid samples (one from each animal) were diluted, 1 : 2, in the buffer included in the Svanova FMDV Antigen detection kit (Boehringer Ingelheim Svanova), and 200 *μ*L of the diluted sample were loaded onto the LFDs as described in Section 2.2. Two LFDs were used per sample. After 30 min, one of the duplicate LFDs was immersed for 15 min in 40 mL of 0.2% citric acid in a 50 mL conical tube and designated as “inactivated”. The corresponding nonsoaked duplicate LFD was designated as “non-inactivated.” LFDs were left to dry before disassembly.

### 2.5. Elution of Viral Particles and RNA Extraction from LFDs

All LFDs were disassembled and processed as described previously [23]. The loading pad, wicking strip, and nitrocellulose band of each LFD were cut into small pieces, vigorously mixed altogether in a microcentritube, and processed for elution, as indicated in Figure 1. All LFDs from Pakistan (20 inactivated labeled D and 20 non-inactivated labeled B) were processed in 560 *µ*L of AVL lysis buffer included in the QIAamp Viral RNA Mini kit (Qiagen, Venlo, The Netherlands). Among the 60 inactivated (labeled C, D, and E) and the 40 noninactivated (labeled A and B) LFDs from Nigeria, 40 (D and E) and 20 (B) were also treated with the same volume of lysis buffer. The remaining LFDs (A and C) were treated with 560 *µ*L of MEM. The same process was applied to LFDs from Turkey. All LFDs from the animal experiment were processed in DMEM.

### 2.6. Virus Isolation

LFBK-*α*V*β*6 or ZZ-R 127 cells were seeded into 24 or 48-well plates to be confluent in 24 hr. The day after, monolayers were washed twice with serum-free cell culture medium. Eluates from one inactivated and one non-inactivated LFD, from each sample from Nigeria, Turkey, and from animal experiments, were inoculated onto cell monolayers (100 *µ*L per well). After 1 hr of adsorption at 37°C under CO_2_, growth medium was added into each well. Cells were then incubated in similar conditions and monitored for the presence of cytopathic effect (CPE). If no CPE was observed after 48 hr, monolayers were freeze–thawed then clarified by centrifugation and used for a second passage on cells under the same conditions.

### 2.7. FMDV Titration by TCID_50_ Assays

The virus content of each vesicular fluid sample from the animal experiment was determined by end-point titration on LFBK-*α*V*β*6 cells. To prevent bacterial and fungal growth, the culture medium was supplemented with 100 U/mL PS, 10 *μ*g/mL gentamicin, and 0.25 *μ*g/mL amphotericin B. Freshly prepared cell suspension was added to the serially diluted samples in 96-well microtiter plates, and the plates were incubated for 3 days at 37°C with 5% CO_2_ and then scored for CPE.

### 2.8. FMDV Detection and Typing by RT-PCR

Viral RNA was extracted from eluates collected from LFDs labeled B, D, and E treated with lysis buffer by using the QIAamp Viral RNA Mini kit (Qiagen) according to the manufacturer's recommendations. Viral RNA was eluted in 60 *µ*L of elution buffer, and 3 *µ*L of SUPERase-RNase Inhibitor (i.e., 60 U) (Invitrogen) was added. A negative control sample (water) was included in each RNA extraction run. Two real-time RT-PCR (rtRT-PCR) assays targeting conserved regions of the FMDV genome (the 3D polymerase coding region and the internal ribosome entry site (IRES)) were used as previously described [27] on RNA extracted from LFDs labeled B, D, and E collected in Turkey and Nigeria and from LFDs labeled D collected in Pakistan. Detection of *β*-actin mRNA was included in both rtRT-PCR assays as an internal control. Alternatively, the 3D polymerase coding region was amplified from RNA extracted from LFDs labeled B collected in Pakistan according to the rtRT-PCR method described in the WOAH manual [5]. Negative (water) and positive (FMDV RNA) controls were included in each rtRT-PCR run.

For FMDV typing, the first strand cDNA was synthesized from 8.9 *µ*L of extracted RNA using the Transcriptor High Fidelity cDNA Synthesis Kit (Sigma-Aldrich), following the manufacturer's instructions. The PCR was carried out on the synthesized cDNA using the One Step RT-PCR kit (Qiagen) without applying the reverse transcription step. From samples derived from Turkey and Pakistan, primers targeting FMDV O, A, and Asia 1 strains circulating in West Eurasia, previously described by Le et al. [28] (*Supplementary 2*), were used in triplicate on cDNA obtained. Alternatively, three sets of primers targeting Asia-1 strains, designed in-house, were also used on cDNA from Pakistan (*Supplementary 2*). From the Nigerian samples, a multiplex PCR targeting O, A, SAT 1, and SAT 2 FMDV strains circulating in West Africa [27] (manuscript in preparation) was performed on cDNA obtained. DNA amplicons were analyzed on a 1.5% agarose electrophoresis gel.

### 2.9. FMDV VP1 Coding Region Amplification, Sequencing, and Sequence Analysis

The VP1 coding region was amplified from the previously obtained cDNA in conventional PCR using the primers listed in *Supplementary 2*, according to the protocol described by Knowles et al. [29]. The One-Step RT-PCR kit (Qiagen) was used on the cDNA without applying the reverse transcription step. Both DNA strands of each amplicon were commercially sequenced (Eurofins Genomics, Ebersberg, Germany) or sequenced in-house with the Sanger dideoxy sequencing method. The VP1 coding sequences were assembled from multiple reads using ContigExpress (DNAstar Inc., Madison, Wisconsin, USA). The nucleotide sequences obtained were then compared with FMDV sequences available in GenBank using the BLASTn online tool (https://blast.ncbi.nlm.nih.gov). Multiple sequence alignment and phylogenetic analyses based on the sequences obtained in our study and homologous FMDV sequences available in GenBank were conducted using MEGA X software [30]. Phylogenetic analyses were conducted by the neighbor-joining method [31], using Tamura 3-parameters [32]. To evaluate the confidence of the tree topology, the bootstrap method was applied with 1,000 replicates [33].

### 2.10. Evaluation of RNA Transfection Protocols and Rescue of Infectious Virus

Five chemical transfection kits—Lipofectamine 2000 (Invitrogen), Lipofectamine 3000 (Invitrogen), Messenger Max (Invitrogen), Trans-IT (Mirus Bio, Madison, WI, USA), and Transfast (Promega, Madison, WI, USA))—were selected based on a literature search (34–37). Multiple ratios of transfection reagents and RNA, listed in *Supplementary 3*, were tested according to manufacturer's instructions on viral RNA freshly extracted from a culture of a laboratory strain (O/IRN/13/2012). Transfection efficiency was evaluated by observing the appearance of CPE on three types of cell monolayers (IBRS-2, ZZ-R 127, and BHK-21). Finally, Lipofectamine 2000 (Invitrogen) and Messenger Max (Invitrogen) were selected and used in parallel on ZZ-R 127 cells, according to the manufacturer's instructions, on all RNA samples extracted from inactivated (labeled D and E) or non-inactivated (labeled B) LFDs. Regarding inactivated LFDs from Turkey and Nigeria, RNA transfection assays were performed in parallel by two laboratories (ANSES processed LFDs labeled D and Boehringer Ingelheim Animal Health processed LFDs labeled E). Regarding LFDs from Pakistan, RNA transfection assays were performed in parallel by two laboratories (ANSES processed inactivated LFDs labeled D and FLI processed non-inactivated LFDs labeled B). At ANSES, positive control samples were treated similarly in parallel (i.e., using FMDV RNA extracted from the reference strain O/IRN/13/2012). Briefly, 2.5 *µ*L of transfection reagent was diluted in 22.5 *µ*L of Opti-MEM (Invitrogen). In parallel, 10 *µ*L of freshly extracted RNA (as described in Section 2.8) were diluted in 15 *µ*L of Opti-MEM. Diluted transfection reagent and diluted RNA were then gently mixed together and incubated for 5 min at room temperature to obtain 50 *µ*L in total. Aliquots (25 *µ*L) were inoculated in duplicate onto confluent ZZ-R 127 cells, seeded 24 hr beforehand in 48-well plates, and incubated at 37°C under 2.5% CO_2_ atmosphere. The appearance of CPE was monitored during the 48 hr. If no CPE was observed after 48 hr, monolayers were freeze–thawed, then clarified by centrifugation, and a second passage was carried out by inoculating the supernatant onto confluent ZZ-R 127 monolayers under conditions described in Section 2.7. If CPE was observed, at the first or second passage, after freeze–thaw and clarification step, rescued viruses were further propagated in confluent ZZ-R 127 cells, seeded 24 hr beforehand in 6-well plates, and then stored at <−70°C.

## 3. Results

### 3.1. FMDV Inactivation on LFDs

The efficacy of the inactivation process in the field was evaluated in Nigeria and Turkey. From each of the 40 positive samples collected and treated in the field, in Turkey (*n* = 20) and Nigeria (*n* = 20), positive results were visible on the LFDs after 10 min of incubation for 96% of the samples collected, and after 30 min for the last 4% (Table 1). From each of these 40 citric acid-inactivated LFDs (labeled C, as in Figure 1) loaded in Nigeria and Turkey, after disassembly, elution steps, and inoculation onto cell monolayers, no CPE was observed after two passages on cells (Table 1). In contrast, after inoculation on cell monolayers of eluates obtained from the corresponding non-inactivated LFDs (labeled A), 38 of the 40 samples produced CPE.

Vesicular fluid from podal vesicles was collected at three or four days postinfection from the six experimentally infected cattle. The FMDV titer in the vesicular fluid ranged from 10^7.6^ to 10^11.0^ TCID_50_/mL (Table 2). Eluates obtained from the acid-treated positive LFDs loaded with these highly infectious vesicular fluids were inoculated onto LFBK-*α*V*β*6 monolayers. No cytotoxicity was observed. No CPE was observed after two passages (Table 2).

**Figure 2:**
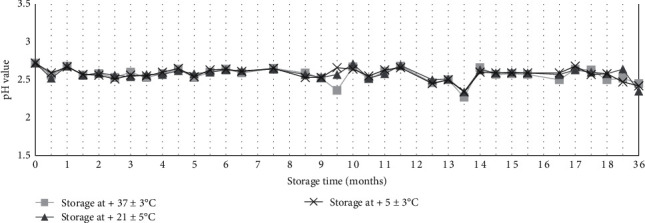
pH values of 0.2% citric acid solutions stored at 5 ± 3, 21 ± 5, or 37 ± 3°C for up to 3 years (i.e., 36 months).

Meanwhile, the stability of the inactivating solution was assessed. Starting from a pH value of 2.72, the pH of the 0.2% citric acid solution was stable after 3 years of storage at 5 ± 3, 21 ± 5, or 37 ± 3°C, with values between 2.27 and 2.72 (Figure 2).

### 3.2. FMDV Genome Detection and Typing

After disassembly and RNA extraction of the 60 inactivated LFDs labeled D (20 from Nigeria, 20 from Turkey, and 20 from Pakistan), FMDV genome was detected for all samples by rtRT-PCR targeting the 3D coding region and the IRES (Table 3). In parallel, similar results have been obtained for the corresponding non-inactivated LFDs labeled B, except for one LFD from Turkey, for which the sample was positive only for the IRES target (data not shown).

As summarized in Table 3, the serotype was successfully identified for nearly 87% of the samples from the inactivated LFDs (labeled D) using the two-step multiplex PCRs. Of 19 samples out of 20 from Nigeria that have been serotyped using this method, serotype O was identified in 15 samples and serotype SAT 2 in four samples. For samples from Turkey, 18 out of 20 were serotyped, and all samples were identified as serotype O. Finally, for samples from Pakistan, 15 out of 20 were serotyped, serotype O was identified in six samples, serotype A in five samples, and serotype Asia 1 in four samples. Serotype identification from inactivated LFDs (labeled D) was confirmed by the typing results obtained on untreated LFDs (labeled B) (data not shown). Antigen ELISA performed in Pakistan on original samples was consistent with the identified serotypes from inactivated and untreated LFDs (data not shown).

### 3.3. VP1 Sequencing and Phylogenetic Analysis

**Figure 3:**
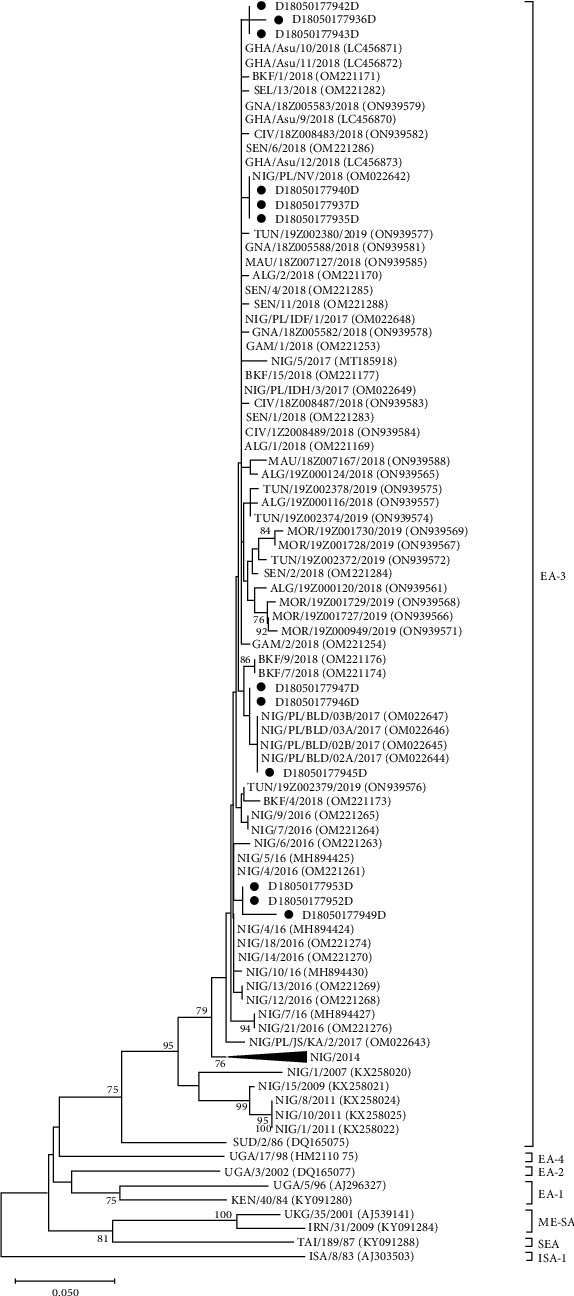
Neighbor-joining phylogenetic tree showing relationships between the nucleotide sequences encoding VP1 of serotype O FMDVs from Nigeria, indicated with a black dot, and sequences available in the GenBank database. The percentages of 1,000 replicates that support each branch node are printed next to the branches, but only bootstrap values >70% are shown. The scale bar indicates nucleotide substitutions per site.

**Figure 4:**
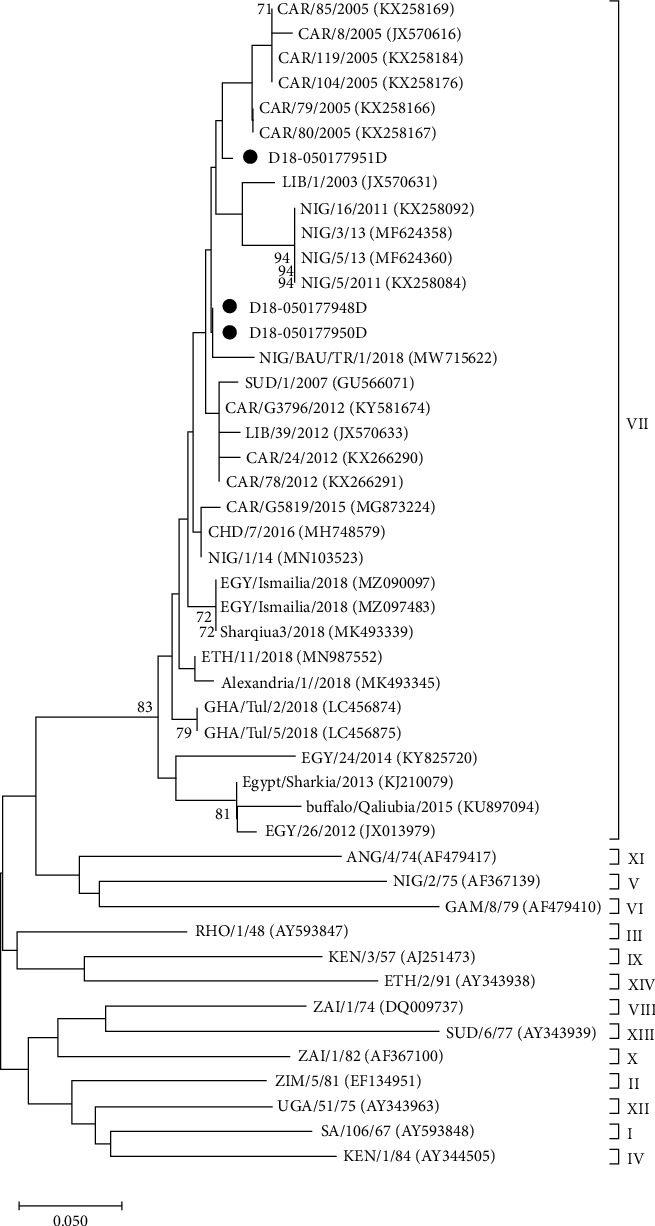
Neighbor-joining phylogenetic tree showing relationships between the nucleotide sequences encoding VP1 of serotype SAT 2 FMDVs from Nigeria, indicated with a black dot, and sequences available in the GenBank database. The percentages of 1,000 replicates that support each branch node are printed next to the branches, but only bootstrap values >70% are shown. The scale bar indicates nucleotide substitutions per site.

**Figure 5:**
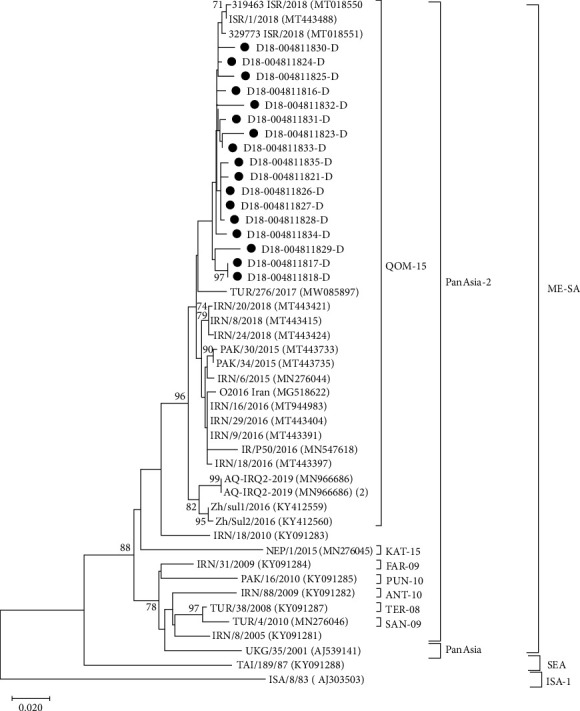
Neighbor-joining phylogenetic tree showing relationships between the nucleotide sequences encoding VP1 of serotype O FMDVs from Turkey, indicated with a black dot, and sequences available in the GenBank database. The percentages of 1,000 replicates that support each branch node are printed next to the branches, but only bootstrap values >70% are shown. The scale bar indicates nucleotide substitutions per site.

Using the LFD samples from Nigeria, Turkey, and Pakistan, Sanger sequencing of the VP1 coding region was attempted (Table 3). VP1 sequences were generated from both the inactivated (labeled D) and untreated (labeled B) LFDs for 12/20 Nigerian samples and for 14/20 Turkish samples (Table 3). The length of the VP1 coding sequence varied between conditions and samples, ranging from full-length and nearly full-length sequences (from 400 to 633 nt) to shorter fragments (from 127 to 271 nt). The seven full VP1 sequences and five partial VP1 sequences recovered from the inactivated LFDs from Nigeria appeared most closely related to FMDV isolates circulating in West and North Africa between 2016 and 2019 and clustered within the FMDV O/EA-3 topotype in the phylogenetic tree (Figure 3). The shorter fragments recovered from three other inactivated LFDs, even though smaller in size, were identified as belonging to the SAT2/VII topotype. These three samples had been collected in the Jos South local government area in the Plateau state of Nigeria. Phylogenetic comparison (Figure 4) revealed that they clustered with FMDV strains circulating in Nigeria in 2018. For samples from Turkey, the 17 sequences from the inactivated LFDs clustered within the FMDV O/ME-SA/PanAsia-2 topotype and its QOM-15 lineage (Figure 5). Phylogenetic comparisons revealed that they were closely related to FMDV strains circulating in Israel in 2018. Regarding samples from Pakistan, four inactivated LFD (D) samples allowed the recovery of partial VP1 sequences, with lengths ranging from 150 to 241 nt (Table 3). These short sequence fragments were identified as most likely belonging to the Asia-1 serotype, ASIA topotype, and Sindh-08 lineage (corresponding to group VII).

### 3.4. Virus Rescue after RNA Transfection

Following the results obtained in a preliminary experiment carried out on the efficiency of RNA transfection conditions, ZZ-R 127 cell monolayers and two of the five transfection kits evaluated (Lipofectamine 2000 and Messenger Max, both Invitrogen) were finally selected for this study (*Supplementary 3*). Both transfection kits were systematically used in parallel.

After transfection of freshly extracted RNA from 100 inactivated LFDs (60 labeled D and 40 labeled E, as shown in Figure 1) collected in the three countries, CPE was observed on transfected cells for nine LFDs, all collected in Nigeria (Table 3). FMD virus was rescued from one RNA sample transfected with Lipofectamine 2000, from four others transfected with Messenger Max, and from four last RNA samples transfected with both reagents. Hence, independently of the transfection reagent applied, nine FMDV strains were rescued by RNA transfection after the inactivation of a positive LFD: three strains of serotype SAT2 and six of serotype O. Among the 60 non-inactivated LFDs (labeled B), after transfection of fresh extracted RNA, FMDV was rescued from 14 samples, all collected in Nigeria (data not shown). Nine of them had also been rescued from RNA extracted from the corresponding inactivated LFD.

## 4. Discussion

Accurate and timely diagnosis of FMD in the case of an outbreak is essential for the rapid implementation of control measures. In addition, full characterization of circulating strains can provide valuable information on the epidemiology and dynamics of the virus as well as for the selection of appropriate vaccines [38]. In the event of an outbreak, it is, therefore, essential to submit field samples quickly to a reference laboratory for diagnosis. However, the rapid shipment of FMDV suspected samples can be a challenge due to their classification as dangerous goods and the need for transportation in dry ice. Indeed, these transport conditions are subjected to strict regulations, banned by some airlines, and can be very expensive. Numerous studies on different devices or processes for the shipment of samples have been carried out. Some are based on the use of FTA cards for FMD [35, 39, 40] or for avian influenza viruses [41]. Other studies were related to the use of LFDs for FMDV [42] or for influenza virus H5N1 [43], which allow virus identification in the field but do not include any virus inactivation step. Additional studies were based on the use of chemical buffers [44–46], including citric acid [47]. In previous work, we reported the development of a safe, easy, and inexpensive protocol, based on the inactivation of FMDV on LFDs, for submitting FMDV samples to diagnostic laboratories [23]. In the present study, we describe the evaluation of this protocol on field samples and confirmed its safety and effectiveness.

The first step of our project was the collection of samples from suspected clinical FMD cases in three endemic countries. In Nigeria and Turkey, epithelium samples were directly and entirely processed in the field. In Pakistan, an extra storage step was applied after the field collection of samples. Probably due to the high virus loads present in fresh clinical samples, all samples tested on LFDs gave positive results.

The second step was to assess the efficiency of FMDV inactivation by citric acid on positive LFDs in Nigeria and Turkey. As expected from our preliminary results for archival samples [23], no CPE was observed after two passages of eluates from treated LFDs on highly sensitive cells (ZZ-R 127 and LFBK-*α*V*β*6), confirming the safety of the citric acid inactivation process directly in the field with fresh samples. To test the efficacy of the inactivation process on very high viral loads, we applied the same inactivation protocol on LFDs loaded with vesicular fluid samples collected from animals experimentally infected with FMDV. Once again, no CPE was observed, even for positive LFDs loaded with fresh vesicular fluid containing up to 10^11.0^ TCID_50_/mL of FMDV. Altogether, these results confirmed that the proposed inactivation protocol is effective and safe. In parallel to this field study, we followed up the pH stability of a 0.2% citric acid solution stored at three different temperatures (+5, +21, and +37°C) for 3 years to mimic potential long-term storage under field conditions. All pH values recorded remained lower than 3. It is well known that the FMDV capsid is highly acid-labile and quickly disassembles at pH values lower than 5 [48]. The citric acid solution with pH under 3 is thus ideal to inactivate this virus. Our results showed the stability of the citric acid solution for up to 3 years after preparation. This supports the possibility of the inclusion of a ready-to-use citric acid solution in LFD kits to ensure the quality of the inactivation solution. Alternatively, this solution could be easily prepared by veterinarians or technicians in the field, provided that they have access to good-quality water and reagents.

After this field study, LFDs were sent to diagnostic laboratories (from Nigeria and Turkey to ANSES and from Pakistan to FLI) to evaluate the performance of the process in terms of FMDV molecular detection and typing. FMDV 3D coding region and IRES sequences were detected by real-time RT-PCR in RNA extracted from all the sixty inactivated LFDs. These results confirmed the sample status determined in the field, using penside tests, and also confirmed that the citric acid treatment did not affect FMDV molecular detection. Accurate characterization of FMDV strains is essential for the implementation of effective control measures, particularly the selection of appropriate vaccines. For almost all samples investigated, molecular typing (by RT-PCR) and VP1 sequencing allowed the identification of the serotype involved. Sequencing results were sufficient to characterize the topotypes, lineages, and sublineages of the FMDV strains and were consistent with the serotypes reported in the literature to circulate in the corresponding areas [12, 49, 50]. There was no significant impact of the LFD inactivation process on the ability to obtain sequence data. For example, VP1-full-length coding sequences for VP1 were generated from five untreated LFDs from Nigeria compared to seven for the inactivated LFDs. We, therefore, believe that, as previously shown in the laboratory, the success of full-length VP1 cDNA amplification depends mainly on the quality and the quantity of the RNA genome recovered from the LFDs.

Virus isolation is an important step in the control of an FMD outbreak using vaccination. The virus isolate can indeed be used to predict vaccine protection in vaccine-matching studies and/or to produce new vaccines [51]. To this end, we attempted to rescue infectious viruses by transfection of RNA extracted from inactivated LFDs. We succeeded in rescuing nine FMDV strains: three of type SAT2 and six of type O, all from inactivated LFDs collected in Nigeria. In parallel, we rescued 14 FMDV strains from non-inactivated LFDs. Overall, for LFDs from Nigeria, we observed around 57% efficiency in virus rescue following RNA transfection. The success rate for RNA transfection could be related to the intrinsic quality of each sample, including the initial amount of viral particles present in the clinical field sample. During an FMD outbreak, the diagnosis is, however, made at the herd level. Thus, the complete characterization of the responsible strain from one infected animal should be sufficient for the selection of the appropriate vaccine and implementation of vaccination to control the outbreak. Using RNA extracted from LFDs loaded in Turkey or Pakistan, inactivated or not, no virus was rescued. Differences in sample processing between the three field campaigns of our study could explain this finding. For example, the quality of water and citric acid used to prepare the inactivation solution could differ, which may impact the stability of the viral RNA on the LFD. The storage of inactivated LFDs for a long time and at a high temperature could also influence virus rescue performance following RNA transfection. Indeed, at most 3 months after loading of fresh clinical samples and the inactivation in the field in Nigeria, LFDs were sent on dry ice to the reference laboratory, where they were stored at −70°C until their further processing. During the entire process, from field sampling in Nigeria to laboratory analysis, which lasted a maximum of 11 months, all samples were kept under cold conditions. Such stable conditions of storage improve RNA preservation despite the long period between LFDs inactivation and their processing in the laboratory, supporting the success of virus rescue following RNA transfection. Since field treatments, storage, and shipment conditions are difficult to control, it could be useful to focus on other ways to improve the efficiency of virus rescue from RNA [52]. Extracted RNA can be concentrated by the use of TRIzol [53]. The introduction of RNA into cells by electroporation could also help to achieve a fairly good rescue rate of FMDV [46]. Such processes and additional cell lines could be evaluated and compared in a further study.

The two main findings of this study are that, first, after citric acid treatment directly on the LFD, no infectious virus was isolated, and second, full-length FMDV RNA was recovered that allowed the rescue of infectious FMDV following transfection. The question of the infectivity of positive sense FMDV RNA arises. According to Keck et al. [40], as noninvasive exposure to RNA did not induce FMD in cattle, the risk of infection from inactivated FMDV samples, even those containing full-length RNA, is deemed negligible. To further reduce the risk of exposure, the traceability of shipments of samples containing inactivated FMDV must obviously be ensured at all times.

Finally, different kinds of alternative procedures have been previously described to preserve and inactivate suspected FMDV samples to simplify their submission to RL, such as the use of FTA cards [35, 54–56] or treatment of tissue samples with commercial buffers such as RNAlater [46], RNA Shield [44] or citrate-phosphate buffer [47]. LFDs have the advantage of allowing first-line diagnostic testing in the field, which can provide a first idea about a clinical FMD suspicion in less than 30 min. Furthermore, LFDs are easy to use, inexpensive but highly specific [57], and the interpretation of the result is simple, with the inclusion of an internal control in each test performed. Moreover, citric acid solution is cheap and accessible (easy to obtain and prepare) in contrast to proprietary commercial buffers such as RNA Shield. To ensure the efficacy of the inactivation step, citric acid solution could be standardized to be ready-to-use and included in the LFD kits. A pH indicator could also be added on the LFD to ensure that the inactivation step was properly completed. Alternatively, inactivated LFDs could probably be shipped to laboratories directly in citric acid solution, but this should be further evaluated to verify RNA integrity after prolonged acid exposure.

In conclusion, the field inactivation protocol described in this study is a safe, fast, and inexpensive way to submit suspected samples of FMDV to RL. Recommendations for the application of this protocol were published in a joint opinion of the EuFMD Standing Technical Committee and the EuFMD Special Committee for Biorisk Management [54]. This will encourage the submission of loaded LFDs and thus promote an active surveillance of circulating FMDV strains for the rapid implementation of control measures.

## Figures and Tables

**Table 1:** Efficacy of virus inactivation process in the field: results obtained after inoculation onto cells of eluates from untreated LFDs or corresponding citric acid-treated LFDs loaded with FMDV samples in Nigeria and Turkey.

Sample origin	Positive result on LFD after 10 min	Positive result on LFD after 30 min	LFD treatment	CPE obtained after inoculation of LFD eluates on cells (after two passages)
Nigeria *n* = 20	20/20	20/20	Untreated LFD “A”	20/20
Turkey *n* = 20	19/20	20/20		18/20
Nigeria *n* = 20	20/20	20/20	Citric acid-treated LFD “C”	0/20
Turkey *n* = 20	18/20	20/20		0/20

CPE, cytopathic effect.

**Table 2:** Results obtained after inoculation onto cells of eluates from citric acid-treated and nontreated positive LFDs loaded with vesicular fluid collected from six experimentally infected cattle.

Cattle number	Collection day (dpi)	Virus titer in vesicular fluid (log_10_ TCID_50_/mL)	Inoculation of LFD eluates onto LFBK-*α*V*β*6 cells	
			After virus inactivation (two passages)	Without inactivation step
190	3	10^10.1^	Negative	CPE
191	3	10^9.8^	Negative	CPE
425	4	10^10.4^	Negative	CPE
508	4	10^8.1^	Negative	CPE
509	4	10^7.6^	Negative	CPE
661	4	10^11.0^	Negative	CPE

dpi, days postinfection; ND, not done; TCID, tissue culture infectious dose; CPE, cytopathic effect.

**Table 3:** FMDV genome detection, VP1 coding sequence characterization, and virus rescue following transfection of RNA extracted from inactivated LFDs and untreated LFDs.

Country	Sample	rtRT-PCR Ct value on inactivated samples		Serotype characterization by two-step multiplex RT-PCR on inactivated samples	VP1 coding sequence generated by Sanger sequencing (nt)		Topotype/lineage characterization	Virus rescue after RNA transfection extracted from inactivated samples	
		3D	IRES		Inactivated	Untreated		Lipofectamine 2000	Messenger Max
Nigeria	D18-050177935	15.3	21.0	O	402	633	O/EA-3	NR	NR
	D18-050177936	15.7	21.9	O	400	/	O/EA-3	NR	NR
	D18-050177937	15.1	22.3	O	633	633	O/EA-3	NR	NR
	D18-050177938	17.6	25.1	O	/	/	NA	NR	NR
	D18-050177939	17.8	24.3	O	/	/	NA	NR	NR
	D18-050177940	16.1	23.3	O	532	142	O/EA-3	Rescued	Rescued
	D18-050177941	14.2	20.7	O	/	143	NA	NR	NR
	D18-050177942	15.3	21.6	O	400	/	O/EA-3	NR	NR
	D18-050177943	17.5	23.4	O	400	/	O/EA-3	NR	Rescued
	D18-050177944	20.3	25.1	Und	/	/	NA	NR	NR
	D18-050177945	18.2	23.2	O	633	532	O/EA-3	NR	Rescued
	D18-050177946	18.5	23.0	O	633	544	O/EA-3	NR	NR
	D18-050177947	17.5	23.1	O	633	633	O/EA-3	Rescued	Rescued
	D18-050177948	14.9	26.0	SAT2	143	271	SAT2/VII	NR	Rescued
	D18-050177949	18.6	26.3	O	470	256	O/EA-3	NR	Rescued
	D18-050177950	18.0	23.7	SAT2	127	642	SAT2/VII	Rescued	Rescued
	D18-050177951	22.4	31.2	SAT2	136	269	SAT2/VII	NR	NR
	D18-050177952	20.2	26.9	O	633	619	O/EA-3	Rescued	NR
	D18-050177953	19.4	26.6	O	633	633	O/EA-3	NR	NR
	D18-050177954	22.0	28.9	SAT2	/	/	NA	Rescued	Rescued

Turkey	D19-004811816	16.2	18.3	O	602	633	O/ME-SA/PanAsia-2^QOM-15^	NR	NR
	D19-004811817	21.1	24.5	O	527	516	O/ME-SA/PanAsia-2^QOM-15^	NR	NR
	D19-004811818	15.5	17.7	O	514	584	O/ME-SA/PanAsia-2^QOM-15^	NR	NR
	D19-004811819	21.7	24.6	Und	/	/	NA	NR	NR
	D19-004811820	17.4	20.4	O	/	633	O/ME-SA/PanAsia-2^QOM-15^	NR	NR
	D19-004811821	17.8	19.8	O	559	633	O/ME-SA/PanAsia-2^QOM-15^	NR	NR
	D19-004811822	26.3	25.5	Und	/	528	O/ME-SA/PanAsia-2^QOM-15^	NR	NR
	D19-004811823	20.4	22.9	O	534	633	O/ME-SA/PanAsia-2^QOM-15^	NR	NR
	D19-004811824	20.9	23.0	O	534	633	O/ME-SA/PanAsia-2^QOM-15^	NR	NR
	D19-004811825	23.0	25.1	O	633	633	O/ME-SA/PanAsia-2^QOM-15^	NR	NR
	D19-004811826	19.0	21.3	O	434	633	O/ME-SA/PanAsia-2^QOM-15^	NR	NR
	D19-004811827	20.0	22.4	O	517	633	O/ME-SA/PanAsia-2^QOM-15^	NR	NR
	D19-004811828	19.4	21.4	O	528	633	O/ME-SA/PanAsia-2^QOM-15^	NR	NR
	D19-004811829	18.2	21.5	O	594	/	O/ME-SA/PanAsia-2^QOM-15^	NR	NR
	D19-004811830	17.2	20.3	O	533	620	O/ME-SA/PanAsia-2^QOM-15^	NR	NR
	D19-004811831	20.6	21.5	O	600	633	O/ME-SA/PanAsia-2^QOM-15^	NR	NR
	D19-004811832	19.9	21.9	O	633	/	O/ME-SA/PanAsia-2^QOM-15^	NR	NR
	D19-004811833	20.3	22.1	O	521	633	O/ME-SA/PanAsia-2^QOM-15^	NR	NR
	D19-004811834	17.2	20.1	O	515	/	O/ME-SA/PanAsia-2^QOM-15^	NR	NR
	D19-004811835	16.9	18.8	O	633	633	O/ME-SA/PanAsia-2^QOM-15^	NR	NR

Pakistan	D19-037158754	20.7	20.4	O	/	ND	NA	NR	NR
	D19-037158755	20.8	19.9	O	/	ND	NA	NR	NR
	D19-037158756	31.6	28.4	Und	/	ND	NA	NR	NR
	D19-037158757	17.2	15.3	Asia	241	ND	Asia-1/ASIA/Sindh-08	NR	NR
	D19-037158758	24.5	21.7	Asia	150	ND	Asia-1/ASIA/Sindh-08	NR	NR
	D19-037158759	22.4	19.1	A	/	ND	NA	NR	NR
	D19-037158760	20.8	18.0	A	/	ND	NA	NR	NR
	D19-037158761	26.8	33.0	Und	/	ND	NA	NR	NR
	D19-037158762	22.1	19.0	A	/	ND	NA	NR	NR
	D19-037158763	21.7	19.2	Asia	241	ND	Asia-1/ASIA/Sindh-08	NR	NR
	D19-037158764	35.3	32.3	Und	/	ND	NA	NR	NR
	D19-037158765	19.4	30.1	A	/	ND	NA	NR	NR
	D19-037158766	24.3	26.7	Und	/	ND	NA	NR	NR
	D19-037158767	20.0	18.1	O	/	ND	NA	NR	NR
	D19-037158768	19.0	15.3	O	/	ND	NA	NR	NR
	D19-037158769	27.7	23.6	Asia	150	ND	Asia-1/ASIA/Sindh-08	NR	NR
	D19-037158770	21.0	31.7	A	/	ND	NA	NR	NR
	D19-037158771	19.1	24.6	O	/	ND	NA	NR	NR
	D19-037158772	24.1	21.8	Und	/	ND	NA	NR	NR
	D19-037158773	20.8	19.7	O	/	ND	NA	NR	NR

Und, undetermined; /, VP1 not amplified; ND, not done; NA, not applicable; NR, not rescued; RT-PCR, reverse transcritpion polymerase chain reaction.

## Data Availability

Research data have been saved and stored on institutional secured servers with backup on a regular base hosted by the respective partners institutions.

## Supplementary Materials

Supplementary 1 Field samples information.

Supplementary 2 Primers used for VP1 coding sequence amplification for typing and sequencing.

Supplementary 3 Comparison of RNA rescue efficiency for five commercial kits on three cell lines.
