# Supplementary material for: Field Evaluation of a Safe, Easy, and Low-Cost Protocol for Shipment of Samples from Suspected Cases of Foot-and-Mouth Disease to Diagnostic Laboratories
Source: Transbound Emerg Dis. 2023 Aug 5;2023:9555213. doi: 10.1155/2023/9555213 (PMC12016716; doi:10.1155/2023/9555213)
Supplement: Supplementary 1 — Field samples information. [file 9555213.f1.docx]

Appendix S1: Field samples information

| Country | Sample reference | Date of collection | City | Local government area / district | State / Province | Species |
| --- | --- | --- | --- | --- | --- | --- |
| Nigeria | D18-050177935 | 2018-04-27 | NC | Barakin ladi | Plateau | Bovine |
| Nigeria | D18-050177936 | 2018-05-01 | NC | Barakin ladi | Plateau | Bovine |
| Nigeria | D18-050177937 | 2018-05-12 | NC | Barakin ladi | Plateau | Bovine |
| Nigeria | D18-050177938 | 2018-05-12 | NC | Barakin ladi | Plateau | Bovine |
| Nigeria | D18-050177939 | 2018-05-12 | NC | Barakin ladi | Plateau | Bovine |
| Nigeria | D18-050177940 | 2018-05-12 | NC | Barakin ladi | Plateau | Bovine |
| Nigeria | D18-050177941 | 2018-05-15 | NC | Riyom | Plateau | Bovine |
| Nigeria | D18-050177942 | 2018-05-15 | NC | Riyom | Plateau | Bovine |
| Nigeria | D18-050177943 | 2018-05-15 | NC | Riyom | Plateau | Bovine |
| Nigeria | D18-050177944 | 2018-05-15 | NC | Riyom | Plateau | Bovine |
| Nigeria | D18-050177945 | 2018-05-17 | NC | Bokkos | Plateau | Bovine |
| Nigeria | D18-050177946 | 2018-05-17 | NC | Bokkos | Plateau | Bovine |
| Nigeria | D18-050177947 | 2018-05-17 | NC | Bokkos | Plateau | Bovine |
| Nigeria | D18-050177948 | 2018-06-21 | NC | Jos South | Plateau | Bovine |
| Nigeria | D18-050177949 | 2018-07-28 | NC | Toro | Plateau | Bovine |
| Nigeria | D18-050177950 | 2018-06-21 | NC | Jos South | Plateau | Bovine |
| Nigeria | D18-050177951 | 2018-06-21 | NC | Jos South | Plateau | Bovine |
| Nigeria | D18-050177952 | 2018-07-28 | NC | Toro | Bauchi | Bovine |
| Nigeria | D18-050177953 | 2018-07-28 | NC | Toro | Bauchi | Bovine |
| Nigeria | D18-050177954 | 2018-07-28 | NC | Toro | Bauchi | Bovine |
| Turkey | D19-004811816 | 2018-06-20 | Iskaniye | Inegöl | Bursa | Bovine |
| Turkey | D19-004811817 | 2018-06-26 | Yeşilyurt | Mihalıçık | Eskişehir | Bovine |
| Turkey | D19-004811818 | 2018-06-26 | Arıkaya | Alpu | Eskişehir | Bovine |
| Turkey | D19-004811819 | 2018-06-27 | Otluk | Mihalıçık | Eskişehir | Bovine |
| Turkey | D19-004811820 | 2018-06-27 | Fatih | Günyüzü | Eskişehir | Bovine |
| Turkey | D19-004811821 | 2018-06-28 | Yunuslar | Gediz | Kütahya | Bovine |
| Turkey | D19-004811822 | 2018-06-29 | Avgan | Uluğbey | Uşak | Bovine |
| Turkey | D19-004811823 | 2018-06-30 | Ergin | Bala | Ankara | Bovine |
| Turkey | D19-004811824 | 2018-07-02 | Efendi | Keskin | Kırıkkale | Ovine |
| Turkey | D19-004811825 | 2018-07-03 | İpekler | Karatay | Konya | Bovine |
| Turkey | D19-004811826 | 2018-07-04 | Ovalıbağ | Çiftlik | Niğde | Bovine |
| Turkey | D19-004811827 | 2018-07-05 | Türkmen | Merkez | Yozgat | Bovine |
| Turkey | D19-004811828 | 2018-07-06 | Kurağıl | Merkez | Kırşehir | Bovine |
| Turkey | D19-004811829 | 2018-07-07 | Karallı | Kızılırmak | Çankırı | Bovine |
| Turkey | D19-004811830 | 2018-07-04 | Acıgöl | Bor | Niğde | Bovine |
| Turkey | D19-004811831 | 2018-06-30 | Davdanlı | Bala | Ankara | Caprine |
| Turkey | D19-004811832 | 2018-07-28 | Doğançayır | Seyitgazi | Eskişehir | Bovine |
| Turkey | D19-004811833 | 2018-07-05 | Müftükışla | Boğazlıyan | Yozgat | Ovine |
| Turkey | D19-004811834 | 2018-07-25 | İkizce | Merkez | Bilecik | Bovine |
| Turkey | D19-004811835 | 2018-07-26 | Kırköy | Uğurludağ | Çorum | Bovine |
| Pakistan | D19-037158754 | 2018-10-21 | Shahbeka | Swat | Khyber Pakhtunkhwa | Bovine |
| Pakistan | D19-037158755 | 2019-01-04 | Sesada | Lower Dir | Khyber Pakhtunkhwa | Buffalo |
| Pakistan | D19-037158756 | 2019-02-12 | Nesata | Charsadda | Khyber Pakhtunkhwa | Buffalo |
| Pakistan | D19-037158757 | 2019-02-17 | Timargara | Lower Dir | Khyber Pakhtunkhwa | Bovine |
| Pakistan | D19-037158758 | 2019-02-24 | Kanju | Swat | Khyber Pakhtunkhwa | Bovine |
| Pakistan | D19-037158759 | 2019-03-19 | Takhtaband | Swat | Khyber Pakhtunkhwa | Bovine |
| Pakistan | D19-037158760 | 2019-03-19 | Takhtaband | Swat | Khyber Pakhtunkhwa | Bovine |
| Pakistan | D19-037158761 | 2019-04-07 | Kanju | Swat | Khyber Pakhtunkhwa | Bovine |
| Pakistan | D19-037158762 | 2019-03-20 | Deolai | Swat | Khyber Pakhtunkhwa | Bovine |
| Pakistan | D19-037158763 | 2019-03-19 | Chamyal | Mansehra | Khyber Pakhtunkhwa | Bovine |
| Pakistan | D19-037158764 | 2017-01-09 | Machob | Ghanche | Gilgit-Baltistan | Bovine |
| Pakistan | D19-037158765 | 2017-01-09 | Machob | Ghanche | Gilgit-Baltistan | Bovine |
| Pakistan | D19-037158766 | 2019-03-26 | Landaky | Swat | Khyber Pakhtunkhwa | Bovine |
| Pakistan | D19-037158767 | 2019-04-16 | Rashakai | Nowshehra | Khyber Pakhtunkhwa | Bovine |
| Pakistan | D19-037158768 | 2019-04-10 | Kalodair | Swabi | Khyber Pakhtunkhwa | Bovine |
| Pakistan | D19-037158769 | NC | Karak | Kara | Khyber Pakhtunkhwa | Bovine |
| Pakistan | D19-037158770 | 2019-04-04 | Naguman | Peshawar | Khyber Pakhtunkhwa | Bovine |
| Pakistan | D19-037158771 | 2017-03-01 | Kanju | Swat | Khyber Pakhtunkhwa | Bovine |
| Pakistan | D19-037158772 | 2017-03-01 | Panr | Swat | Khyber Pakhtunkhwa | Bovine |
| Pakistan | D19-037158773 | 2017-03-01 | Nazir Bagh | Peshawar | Khyber Pakhtunkhwa | Buffalo |

NC: not communicated

Legend: Information about the sixty epithelium samples collected from suspected clinical cases of FMD are listed above. Twenty samples from Nigeria were collected between April and July 2018, twenty samples from Turkey June and July 2018, and twenty samples from Pakistan from January 2017 to April 2019. The location (country, city, local government area or district, and state or province), date of sampling and host species are indicated.
