# Supplementary material for: Field Evaluation of a Safe, Easy, and Low-Cost Protocol for Shipment of Samples from Suspected Cases of Foot-and-Mouth Disease to Diagnostic Laboratories
Source: Transbound Emerg Dis. 2023 Aug 5;2023:9555213. doi: 10.1155/2023/9555213 (PMC12016716; doi:10.1155/2023/9555213)
Supplement: Supplementary 2 — Primers used for VP1 coding sequence amplification for typing and sequencing. [file 9555213.f2.docx]

Appendix S2: Primers used for VP1 coding sequence amplification for typing and sequencing

| Primers F and R | Sequences (5’→3’) | Application | FMDV serotype targeted | References |
| --- | --- | --- | --- | --- |
| O-1C-244-F | GCAGCAAAACACATGTCAAACACCTT | Sequencing | O | (Ayelet et al., 2009) |
| A-1C-562-F | TACCAAATTACACACGGGAA | Sequencing | A |  |
| EUR-2B-52-R | GACATGTCCTCCTGCATCTGGTTGAT | Sequencing | O, A, Asia |  |
| SAT2-1C-445-F | TGGGACACMGGIYTGAACTC | Sequencing | SAT2 |  |
| SAT-2B-208-R | ACAGCGGCCATGCACGACAG | Sequencing | SAT-1-2-3 |  |
| VN-O-F | AGATTTGTGAAAGTDACACCA | Typing | O | (Le et al., 2011) |
| VN-A-F | CTTGCACTCCCTTACACCGCG | Typing | A |  |
| VN-Asia-F | GCGSTHRYYCACACAGGYCCGG | Typing | Asia |  |
| VN-VP1-R | CATGTCYTCYTGCATCTGGTT | Typing | O, A, Asia |  |
| PAK_Asia_F_1 | GAGTCAGCAGACCCAGTCAC | Typing | Asia | Unpublished,  primers designed in-house |
| PAK_Asia_R_1 | ACCTGTCGAGAACGAAGCTG | Typing | Asia |  |
| PAK_Asia_F_5 | CTTGCCCACCTCCTTCAACT | Typing | Asia |  |
| PAK_Asia_R_5 | GGGTGCGATGATCTCCTGTT | Typing | Asia |  |
| PAK_Asia_F_10 | TAAGACTGCCTTGGACTGCC | Typing | Asia |  |
| PAK_Asia_R_10 | CGTACGCTGTTTTCCCGTTG | Typing | Asia |  |

Legend: The name and the sequence (5’→3’) of forward (F) and reverse (R) primers used to amplify the VP1 coding sequence for typing and sequencing application are listed above. The FMDV serotype targeted by each primer is specified.
