# Supplementary material for: Field Evaluation of a Safe, Easy, and Low-Cost Protocol for Shipment of Samples from Suspected Cases of Foot-and-Mouth Disease to Diagnostic Laboratories
Source: Transbound Emerg Dis. 2023 Aug 5;2023:9555213. doi: 10.1155/2023/9555213 (PMC12016716; doi:10.1155/2023/9555213)
Supplement: Supplementary 3 — Comparison of RNA rescue efficiency for five commercial kits on three cell lines. [file 9555213.f3.docx]

Appendix S3: Comparison of RNA rescue efficiency for five commercial kits on three cell lines

| Transfection kit | Volume of transfection reagents (µL)  + volume of total RNA extracted (µL) | Appearance of cytopathic effect in cell monolayers at 24 hours post-transfection | | |
| --- | --- | --- | --- | --- |
|  |  | IBRS-2 | ZZ R-127 | BHK-21 |
| Lipofectamine 2000 (Invitrogen) | 0.5 µL reagent + 0.25 µg RNA | + | ++ | - |
|  | 0.75 µL reagent + 0.25 µg RNA | ++ | +++ | - |
|  | 1 µL reagent + 0.25 µg RNA | ++ | +++ | - |
|  | 1.25 µL reagent + 0.25 µg RNA | ++ | +++ | - |
|  | 0.5 µL reagent + 0.025 µg RNA | - | + | - |
|  | 0.75 µL reagent + 0.025 µg RNA | - | ++ | - |
|  | 1 µL reagent + 0.025 µg RNA | - | ++ | - |
|  | 1.25 µL reagent + 0.025 µg RNA | - | ++ | - |
| Lipofectamine 3000  (Invitrogen) | 0.4 µL reagent + 0.25 µg RNA | - | + | - |
|  | 0.4 µL reagent + 0.25 µg RNA + 0.5 µL P3000 | +/- | ++ | - |
|  | 0.8 µL reagent+ 0.25 µg RNA | + | ++ | - |
|  | 0.8 µL reagent+ 0.25 µg RNA + 0.5 µL P3000 | + | ++ | - |
|  | 0.4 µL reagent + 0.025 µg RNA | - | +/- | - |
|  | 0.4 µL reagent + 0.025 µg RNA + 0.5 µL P3000 | - | + | - |
|  | 0.8 µL reagent + 0.025 µg RNA | - | +/- | - |
|  | 0.8 µL reagent + 0.025 µg RNA + 0.5 µL P3000 | - | +/- | - |
| Messenger MAX  (Invitrogen) | 0.5 µL reagent + 0.25 µg RNA | +++ | ++ | - |
|  | 0.8 µL reagent + 0.25 µg RNA | +++ | ++ | - |
|  | 1 µL reagent + 0.25 µg RNA | +++ | +++ | - |
|  | 1.3µL reagent + 0.25 µg RNA | Toxicity | +++ | - |
|  | 0.5 µL reagent + 0.025 µg RNA | - | ++ | - |
|  | 0.8 µL reagent + 0.025 µg RNA | - | ++ | - |
|  | 1 µL reagent + 0.025 µg RNA | - | ++ | - |
|  | 1.3µL reagent + 0.025 µg RNA | - | ++ | - |
| Trans-IT  (Mirus Bio) | 0.5 µL reagent + 0.5 µL Booster + 0.25 µg RNA | +++ | ++ | +/- |
|  | 0.5 µL reagent + 1 µL Booster + 0.25 µg RNA | +++ | ++ | +/- |
|  | 1 µL reagent + 0.5 µL Booster + 0.25 µg RNA | +++ | + | +/- |
|  | 1 µL reagent + 1 µL Booster + 0.25 µg RNA | +++ | + | +/- |
|  | 0.5 µL reagent + 0.5 µL Booster + 0.025 µg RNA | +/- | +/- | - |
|  | 0.5 µL reagent + 1 µL Booster + 0.025 µg RNA | +/- | +/- | - |
|  | 1 µL reagent + 0.5 µL Booster + 0.025 µg RNA | +/- | +/- | - |
|  | 1 µL reagent + 1 µL Booster + 0.025 µg RNA | +/- | +/- | - |
| Transfast  (Promega) | 0.25 µL reagent + 0.25 µg RNA | ++ | ++ | +/- |
|  | 0.5 µL reagent + 0.25 µg RNA | ++ | ++ | +/- |
|  | 0.75 µL reagent + 0.25 µg RNA | ++ | ++ | +/- |
|  | 0.025 µL reagent + 0.025 µg RNA | +/- | Toxicity | - |
|  | 0.05 µL reagent + 0.025 µg RNA | +/- | Toxicity | - |
|  | 0.075 µL reagent + 0.025 µg RNA | +/- | Toxicity | - |

-=no cytopathic effect; +/-=some cells rounded; +=half of cells lysed; ++= majority of cells lysed; +++=complete cytopathic effect

Legend: Five chemical transfection kits – Lipofectamine 2000, Lipofectamine 3000, Messenger Max (Invitrogen, Carlsbad, CA, USA), Trans-IT (Mirus Bio, Madison, WI, USA), and Transfast (Promega, Madison, WI, USA) – were selected based on a literature search (Avci-Adali et al., 2014; Biswal, Subramaniam, Ranjan, & Pattnaik, 2016; Gonzalez, Pfannes, Brazas, & Striker, 2007; Oh & Kessler, 2018). Multiple ratios of transfection reagents and RNA, were tested according to manufacturer’s instructions on viral RNA freshly extracted from a culture of a FMDV laboratory strain. Transfection efficiency was evaluated by observing the appearance of cytopathic effect on three types of cell monolayers (IBRS-2, ZZ-R 127 and BHK-21).
